# Detecting Plant Stress Using Thermal and Optical Imagery From an Unoccupied Aerial Vehicle

**DOI:** 10.3389/fpls.2021.734944

**Published:** 2021-10-27

**Authors:** Bonny Stutsel, Kasper Johansen, Yoann M. Malbéteau, Matthew F. McCabe

**Affiliations:** Hydrology, Agriculture and Land Observation, Water Desalination and Reuse Center, King Abdullah University of Science and Technology, Thuwal, Saudi Arabia

**Keywords:** unoccupied aerial vehicle, unmanned aerial vehicle, thermal infrared, salt tolerance, phenotyping, tomato, plant stress, accessions

## Abstract

Soil and water salinization has global impact on the sustainability of agricultural production, affecting the health and condition of staple crops and reducing potential yields. Identifying or developing salt-tolerant varieties of commercial crops is a potential pathway to enhance food and water security and deliver on the global demand for an increase in food supplies. Our study focuses on a phenotyping experiment that was designed to establish the influence of salinity stress on a diversity panel of the wild tomato species, *Solanum pimpinellifolium*. Here, we explore how unoccupied aerial vehicles (UAVs) equipped with both an optical and thermal infrared camera can be used to map and monitor plant temperature (T_p_) changes in response to applied salinity stress. An object-based image analysis approach was developed to delineate individual tomato plants, while a green–red vegetation index derived from calibrated red, green, and blue (RGB) optical data allowed the discrimination of vegetation from the soil background. T_p_ was retrieved simultaneously from the co-mounted thermal camera, with T_p_ deviation from the ambient temperature and its change across time used as a potential indication of stress. Results showed that T_p_ differences between salt-treated and control plants were detectable across the five separate UAV campaigns undertaken during the field experiment. Using a simple statistical approach, we show that crop water stress index values greater than 0.36 indicated conditions of plant stress. The optimum period to collect UAV-based T_p_ for identifying plant stress was found between fruit formation and ripening. Preliminary results also indicate that UAV-based T_p_ may be used to detect plant stress before it is visually apparent, although further research with more frequent image collections and field observations is required. Our findings provide a tool to accelerate field phenotyping to identify salt-resistant germplasm and may allow farmers to alleviate yield losses through early detection of plant stress *via* management interventions.

## Introduction

The area of agricultural land impacted by salinization and sodification is increasing globally, with more than 50% of arable land predicted to be affected by 2050 (Wang et al., 2003; Jamil et al., 2011). Concurrently, it is anticipated that crop production will need to more than double to meet the demands of a projected 10 billion people by 2050 (Ray et al., 2013). Furthermore, increasing affluence and shifting diets toward greater meat consumption mean that without improvements in productivity, water consumption in agriculture will increase by a further 70–90% over the same period (Molden, 2013; Pittock et al., 2016). Global freshwater supplies are under extreme pressure, with agricultural production already accounting for more than two-thirds of freshwater use (Famiglietti, 2014; Brauman et al., 2016; Pastor et al., 2019). Therefore, irrigation with brackish water presents as an enticing option, as the targeted application of water is an effective way to close the yield gap (Licker et al., 2010; Mueller et al., 2012). The identification and breeding of cultivars with increased resilience to salt stress would provide an effective twofold solution to ensuring future food security by enabling production on marginal land and the potential to irrigate with brackish water (Morton et al., 2018).

Salt stress in plants results in complex physiology and morphometric changes that occur in two distinct phases (Munns and Tester, 2008). The first phase occurs rapidly (minutes to days) as the plant responds to the buildup of salt in the roots, which leads to reduced osmotic potential and hence water uptake. This phase is referred to as ion-independent and causes stomatal closure and a reduction in new shoot growth. The second ionic phase occurs more slowly (days to weeks) once salt concentration in the leaves reaches cytotoxic levels, resulting in senescence of mature leaves (Munns and Tester, 2008; Isayenkov and Maathuis, 2019). A plant’s response to salt stress also varies with the growing environment (Maas, 1993), making field trials necessary to assess stress in agronomically important traits such as yield quantity and quality. Despite focused research efforts, there has been little progress in identifying salt-tolerant genes. Researchers attribute this lack of progress to the genetic complexity of salt tolerance (Morton et al., 2018) and the limitations of manual field phenotyping (Araus and Cairns, 2014). New tools and approaches are required to bridge this phenotype-to-genotype divide (McCabe and Tester, 2021).

Recent advances in remote sensing technologies offer a means to overcome some of the limitations of traditional field phenotyping. Unpiloted aerial vehicles (UAVs) mounted with multispectral, hyperspectral, and thermal sensors have proven particularly useful for phenotyping due to their ability to capture plant data at unprecedented spatial (sub-cm), temporal (on-demand), and spectral resolutions. Laborious and often subjective manual measurements of plant phenotypic traits can now be augmented by consistent information derived for an entire field in a single flight and with repeatability across the growth cycle (Araus and Cairns, 2014; Holman et al., 2016). For example, UAV-captured data can provide insights on plant nitrogen status (Perry et al., 2018), height (Ziliani et al., 2018), biomass (Bendig et al., 2014; Johansen et al., 2020), and temperature (Deery et al., 2016; Malbéteau et al., 2018) at the field scale and on demand, which is accelerating field screening and selection of germplasm for agronomically important traits to guide breeding programs and optimize commercial cultivars (Hickey et al., 2019).

The last decade has seen a rapid expansion in the application of UAVs for field phenotyping (Yang et al., 2017; Xie and Yang, 2020). However, applications of UAV-based sensing in salinized environments for rapid identification of salt-tolerant germplasm are relatively unexplored, despite research showing that wild-growing relatives (e.g., *Solanum pimpinellifolium*) of cultivated crops (e.g., *Solanum lycopersicum*) have increased salt tolerance (Zuriaga et al., 2009; Rao et al., 2013; Bolger et al., 2014; Razali et al., 2018). Johansen et al. (2019, 2020) addressed this gap by assessing phenotypic traits, including tomato plant area, plant cover, growth rate, condition, biomass, and yield from UAV-based multispectral imagery to discriminate plant performance under salt stress and control conditions. They identified distinct differences in phenotypic traits between control and salt-treated plants and found the traits suitable for identifying most of the highest yield-producing plant accessions. They also incorporated these traits into a random forest approach to predicting yield before harvest. Overall, their results indicated that salt tolerance is evident in many phenotypic expressions and is best discriminated from other abiotic and biotic stresses by incorporating UAV measurements of multiple traits.

Extending on these prior studies, we investigate the collection of plant temperature measurements (T_p_) derived from UAV-based thermal infrared (TIR) cameras to screen for salt stress. T_p_ is commonly used as a surrogate for stomatal conductance, as stomatal closure results in reduced transpiration, which in turn leads to an increase in T_p_ (Tanner, 1963; Jones, 2013). However, TIR-based T_p_ is also influenced by environmental factors such as net radiation, vapor pressure deficit (VPD), and wind speed (Jackson et al., 1988). Therefore, researchers commonly use T_p_ measurements in combination with air temperature (T_a_) for TIR indices such as the crop water stress index (CWSI) (Idso et al., 1981; Jackson et al., 1981) to normalize data and compare plant stress across multiple days. T_p_ and its use *via* the CWSI have been explored in broad-acre crops (Bian et al., 2019; Gracia-Romero et al., 2019; Zhang et al., 2019), tree crops (Gonzalez-Dugo et al., 2012, 2014; Park et al., 2017), and vineyards (Baluja et al., 2012; Bellvert et al., 2016; Sepúlveda-Reyes et al., 2016; Kustas et al., 2018). From an analysis of the recent literature, an examination of T_p_ retrievals in annual vegetable crops seems to be limited to potato plants (Rud et al., 2012, 2014). The ability to detect salinity-induced stress in tomato plants *via* remotely sensed T_p_ in the initial ion-independent phase would be particularly helpful in providing an early detection method of stress before changes in plant color or shape occur.

Using remotely sensed T_p_ as an indicator of stress requires its accurate retrieval from UAV TIR imagery, which remains challenging (Aragon et al., 2020; Döpper et al., 2020; Perich et al., 2020). First, UAV TIR cameras use lightweight uncooled microbolometers, making them prone to thermal drift (Gómez-Candón et al., 2016; Mesas-Carrascosa et al., 2018; Döpper et al., 2020). Second, the impact of vignetting and dead pixels in the focal plane array needs to be accounted for (Kelly et al., 2019; Aragon et al., 2020). Third, the methods used to generate the orthomosaic from which T_p_ is retrieved will also influence the apparent temperature (Perich et al., 2020). Fourth, shadowing within the plant canopy can lead to large temperature differences between sunlit and shaded components, which may require consideration (Jones et al., 2002). Fifth, the soil background temperature integration can bias the retrieved T_p_ (Jones and Sirault, 2014). Finally, the sensitivity of T_p_ to environmental variation means that weather changes such as wind speed, wind direction, or cloud cover across a flight can introduce uncertainty (Maes et al., 2017).

Overcoming the low radiometric accuracy of UAV-based TIR cameras has led to the development of laboratory-based and vicarious calibration procedures to improve temperature retrievals (see Jensen et al., 2014; Khanal et al., 2017; Maes et al., 2017; Ribeiro-Gomes et al., 2017; Torres-Rua, 2017; Aragon et al., 2020). Even though calibration procedures are employed, research to date demonstrates the need to carefully consider how data are captured, processed, and ultimately used to retrieve T_p_. Researchers have employed many methods to identify vegetation pixels from which to retrieve T_p_ in coarse TIR imagery. Researchers interested in bulk canopy temperature have previously used simple polygons to delineate plots (Deery et al., 2016; Gracia-Romero et al., 2019; Perich et al., 2020). However, this method only works for crops with canopy closure, which precludes the impact of the background soil temperature on T_p_ retrievals. Therefore, TIR imagery is commonly co-registered to red, green, and blue (RGB), multispectral, or hyperspectral imagery so that vegetation indices or classification algorithms can be applied to identify pixels representing vegetation (Rud et al., 2014; Zhang et al., 2019; Maimaitijiang et al., 2020). To prevent reliance on other data sources, a number of approaches have been developed based solely on TIR imagery for T_p_ retrieval (Meron et al., 2010, 2013; Cohen et al., 2017; Park et al., 2017; Bian et al., 2019). Often, such approaches delineate canopy extent using edge detection methods, from which they can then retrieve T_p_ from pixels.

For a method to be adopted in precision agriculture workflows, it needs to be farmer-friendly and as straightforward as possible (Cohen et al., 2017). Based on the reviewed literature, there is currently a significant knowledge gap and disconnect between obtaining and extracting UAV-based TIR information and then ensuring this information can be translated into meaningful biological understanding at the individual plant scale (Kellner et al., 2019). Our research presents an approach for retrieving T_p_ from UAV-based TIR and RGB imagery, with an experimental focus on a diversity panel of tomato plants undergoing drip-irrigation in both control and salt water conditions. The retrieved T_p_ is interrogated to understand its response to plants experiencing salt stress and establish if TIR-based indices can identify: differences in plant stress between control and salt-treated plants, and the optimum time during the growing season to detect plant stress using multi-temporal UAV-based TIR data.

## Materials and Methods

### Description of Study Site

**Figure 1:**
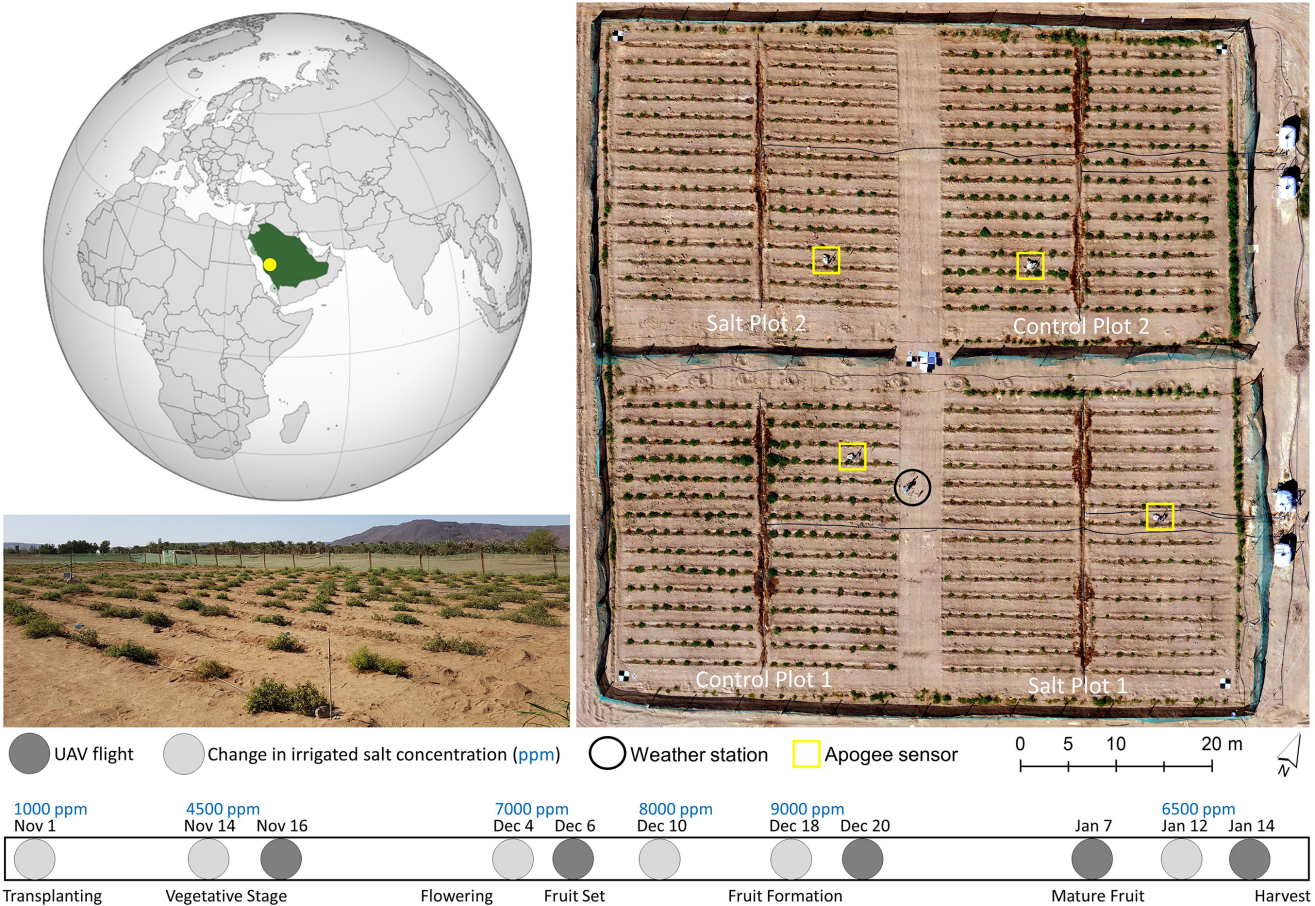
**Left:** The location of the tomato field experiment and a field photograph of Salt Plot 2 at the King Abdulaziz University Agricultural Research Station, Hada Al-Sham, Saudi Arabia (21° 47ʹ48ʺN, 39° 43ʹ35ʺ E). **Right:** A UAV-derived orthomosaic of the site captured on January 14, 2018, showing the trial layout. Bottom: The timing of UAV flights, tomato phenological stages, and concentrations of salt in parts per million (ppm) in the water used to irrigate the salt-treated plots across the growing season.

The study took place during the 2017–2018 growing season (November–January) at a field located within the King Abdulaziz University Agricultural Research Station in Hada Al-Sham, Saudi Arabia (21° 47ʹ48ʺN, 39° 43ʹ35ʺE, Figure 1). The field was divided into four separate plots, each approximately 40m x 40m, with 15 rows of 20 tomato plants. Two plots were established as controls, with freshwater irrigation (approx. 900–1,000ppm NaCl). The other two plots were irrigated twice daily (except Fridays) with saline water of increasing concentrations (Figure 1). In developing the diversity panel, 200 accessions (199 wild *Solanum pimpinellifolium* and one commercial *S. lycopersicum*) were screened for salt tolerance *via* randomized planting of three replications of each accession for each treatment (i.e., three salt-treated and three control plants per accession, producing a total of 1,200 plants). At the beginning of November, 1,200 seedlings were transplanted into the field (after 1month of greenhouse growth), with harvesting taking place between 16 and 26 January (Figure 1). Additional details of the site and trial design information can be found in Aragon et al. (2020)) and Johansen et al. (2019). The focus of this study was to understand whether TIR data can identify differences in plant stress between control and salt-treated *Solanum pimpinellifolium* plants.

A weather station was installed toward the middle of the field (Figure 1) to collect meteorological data throughout the growing season. T_a_ and relative humidity (RH) were recorded every minute at 2.3m above ground level (AGL) using an HMP155 humidity and temperature probe (Vaisala, Helsinki, Finland), from which the VPD was calculated (May et al., 2008). Wind speed and direction were also recorded every minute at 2.2m AGL with a WindSonic anemometer (Gill, Hampshire, United Kingdom). Meteorological data were augmented by four distributed stations in each of the plots that measured point-scale thermal infrared temperature *via* an Apogee radiometer (SI-111, Apogee, Logan, United States), which facilitates interpretation of the UAV-collected TIR data (see locations in Figure 1). The Apogee sensors were installed in each plot approximately 1m above a plant, representing a footprint of around 0.40m^2^. As our study occurred in an arid desert environment, sandstorms impacted the site on December 8 and 16, 2017, and January 4 and 8–10, 2018. To combat the impact of the sandstorms on results, field staff washed the plants with non-saline water after each event.

### Thermal Infrared and Optical RGB Data Collection and Processing

#### Thermal Infrared Image Collection and Processing

TIR images were captured using a gimbal-stabilized FLIR Tau 2 core with a ThermalCapture 2.0 capture system (TeAx, Wilnsdorf, Germany) mounted on a DJI Matrice 100 quadcopter (Da Jiang Innovations, Shenzhen, China). The camera has a broadband spectral range across 7.5–13.5 um with a resolution of 640×512 pixels and a focal length of 13mm. Manufacturer guidelines indicate temperature retrievals with a specified accuracy of ±5°C and sensitivity of 0.04°C. Flying height was 13m AGL at a speed of 2m.s^−1^ for a total flight duration of approximately 17min, with flight times shown in Table 1. The imagery was collected from a nadir view, with around 60% sidelap and 93% forward overlap. Five large circular aluminum trays that can be easily distinguished in the TIR data (due to their low emissivity) were deployed at both the center and each corner of the field as ground control points (GCPs) (Figure 1). Each GCP’s location was surveyed using a Leica AS10 Real-Time Kinematic Global Navigation Satellite System and base station (Leica Geosystems, St. Gallen, Switzerland).

**Table 1:** UAV data collection date, start time and coincident mean air temperature (T_a_), relative humidity (RH), wind speed (WS), and vapor pressure deficit (VPD) for the 17-min flights.

UAV Flight Date	Start Time	Ta (°C)	RH (%)	WS (ms^−1^)	VPD (kPa)
November 16, 2017	13:33	32.83	38.78	4.02	3.05
December 06, 2017	11:00	32.48	22.89	2.85	3.77
December 20, 2017	11:56	32.14	14.39	2.29	4.11
January 07, 2018	12:42	29.79	15.85	1.44	3.53
January 14, 2018	12:47	30.11	27.76	2.43	3.09

Before deploying the TeAx 640 camera, a temperature-dependent radiometric calibration matrix was applied to correct ambient temperature dependency, vignette effects, and other non-uniformity noise (Aragon et al., 2020). The multilinear regression matrix from Aragon et al. (2020) was applied to the collected thermal data before subsequent processing. In this correction, the mean T_a_ acquired during each flight was used for the temperature-dependent radiometric calibration to remove any influence of ambient temperature dependency. Geo-referencing and orthorectification of the TIR imagery were performed using Agisoft PhotoScan (Agisoft LLC, St. Petersburg, Russia). Before image alignment and scene reconstruction based on matched feature points, the calibrated radiance values were linearly stretched to the full dynamic range to improve feature identification. The image alignment step also performs a bundle adjustment to estimate the camera positions, orientations, and lens calibration parameters. Hence, to recalculate the camera positions, the self-calibrating bundle adjustment computes three-dimensional point clouds from which thermal orthophotos were built (Malbéteau et al., 2021).

For each of the five UAV campaigns, approximately 150 individual geo-referenced and orthorectified images were collected across each of 18 flight lines. Due to the forward overlap of 93% and the near-identical acquisition time of neighboring overlapping images, an averaging approach was applied to each pixel in the overlapping areas of each swath. The averaging method was applied to each swath due to the rapid changes in surface temperature and the impact of environmental conditions on the uncooled (unstabilized) sensor, which is often a significant challenge for UAV-based TIR processing (Aragon et al., 2020). To alleviate the influence of flight orientation relative to the wind direction and to ensure normalization of neighboring swaths, a flight direction correction method was also applied. The correction method normalized the pixel values within the neighboring swaths by assuming a 0°C difference between the overlapping (60% sidelap) areas. Initially, the first swath of the flight survey was used for correcting the second swath. Then, the second corrected swath was used for correcting the third swath and so forth. Adjusting the temperatures of each swath one by one and starting with the first swath of the flight survey ensured that all swaths were also corrected for temperature variability experienced during the 17min of flight time (Malbéteau et al., 2021). The normalization process of individual swaths allowed them to be merged to form an orthomosaic.

#### Optical RGB Image Collection and Processing

RGB data were collected with a Zenmuse X3 camera (Dà-Jiāng Innovations, Shenzhen, China) concurrently with the TIR data, except on December 6, 2017, when RGB data were collected at 11:44 (approximately 44min after the TIR data collection). The RGB image collection occurred with 82% sidelap and 93% along-track overlap, with a photograph captured every 3s. All UAV data were collected under clear sky conditions and close to solar noon to reduce sun angle impacts on the RGB data (Table 1). RGB imagery was processed in Agisoft PhotoScan (Agisoft LLC, St. Petersburg, Russia) to construct a geometrically corrected orthomosaic, which was then radiometrically corrected using calibration panels and the empirical line method (Smith and Milton, 1999). Additional information regarding the collection, processing, and calibration of the RBG imagery is outlined in Johansen et al. (2019).

**Figure 2:**
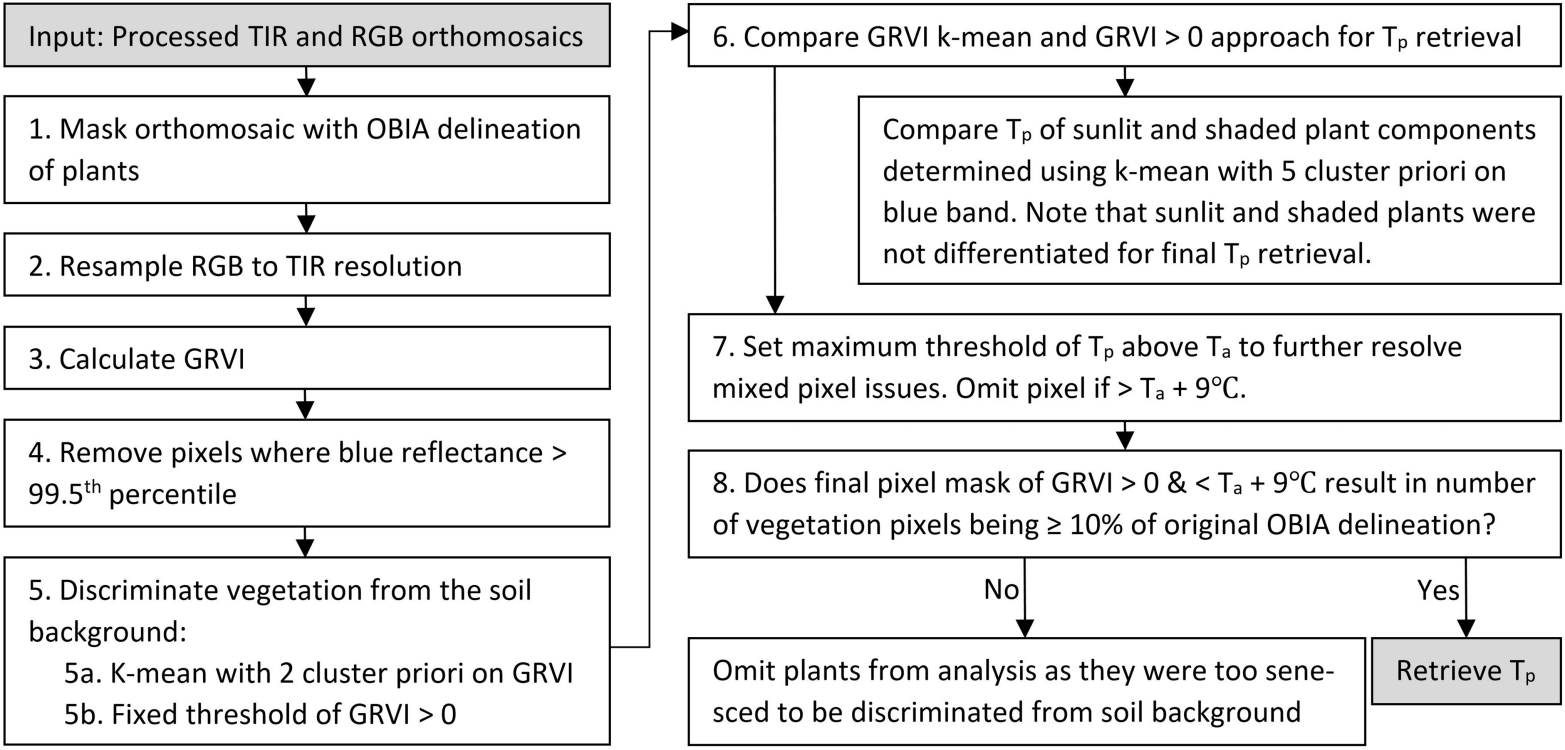
Workflow to retrieve plant temperature (T_p_) of green vegetation from the thermal infrared (TIR) orthomosaic using an object-based image analysis (OBIA) delineation of the red, green, and blue (RGB) image data, k-mean classification, green–red vegetation index (GRVI) thresholding, and air temperature (T_a_).

The processed RGB orthomosaics had a GSD of 0.005m. The RGB orthomosaics were resampled to the same resolution as the TIR orthomosaics (0.015m) using nearest-neighbor resampling in the rasterio.warp module (Gillies et al., 2013). The resampling was undertaken to ensure that the RGB data could be used to determine each plant’s extent for T_p_ retrieval from the TIR data (Figure 2). To ensure accurate co-registration of the TIR and RGB datasets, the RGB orthomosaics were manually geo-referenced in QGIS (QGIS Development Team, 2021) to the TIR data using the five GCPs with a polynomial transformation, resulting in a mean square error between the centers of each GCP across all campaigns of approximately 0.01m.

### Retrieving Plant Temperature From the Thermal Infrared Orthomosaics

An object-based image analysis (OBIA) approach was applied to the RGB orthomosaics to identify each plant’s extent in the TIR orthomosaic (Figure 2, Step 1). A full description of the workflow used to create the OBIA RGB delineations can be found in Johansen et al. (2019). In order to omit pixels within the delineated plants that were associated with white identification tags (attached to individual plants), pixels with blue reflectance above the 99.5th percentile were removed. Next, green vegetation was discriminated within the delineated objects by applying a k-means clustering to the green–red vegetation index (GRVI) (Motohka et al., 2010). The GRVI was calculated as per Eq. 1 using the collected RGB data, as this index produced good results in Johansen et al. (2019; Figure 2, Step 5a). We applied a k-mean unsupervised approach run with two clusters, k-means++ initialization, ten different centroid seeds, and a maximum iteration of 300 in the scikit-learn package of the Python 3.5 software (Pedregosa et al., 2011). We set two clusters since the plants had already been delineated with the OBIA approach, and we were merely interested in discriminating vegetation from the sandy background, which had distinct spectral characteristics. For the classification of vegetation, a threshold value of GRVI > 0 was also used (Motohka et al., 2010). The distribution of temperature for vegetation classified with both the k-means approach and the GRVI threshold was subsequently compared to determine the most suitable approach (Figure 2, Step 6).


$$\mathrm{Green-red}\;\mathrm{vegetation index}\;\left(\mathrm{GRVI}\right)=\frac{Green-\mathrm{red}}{Green+\mathrm{red}\;}\;$$ (1)

**Figure 3:**
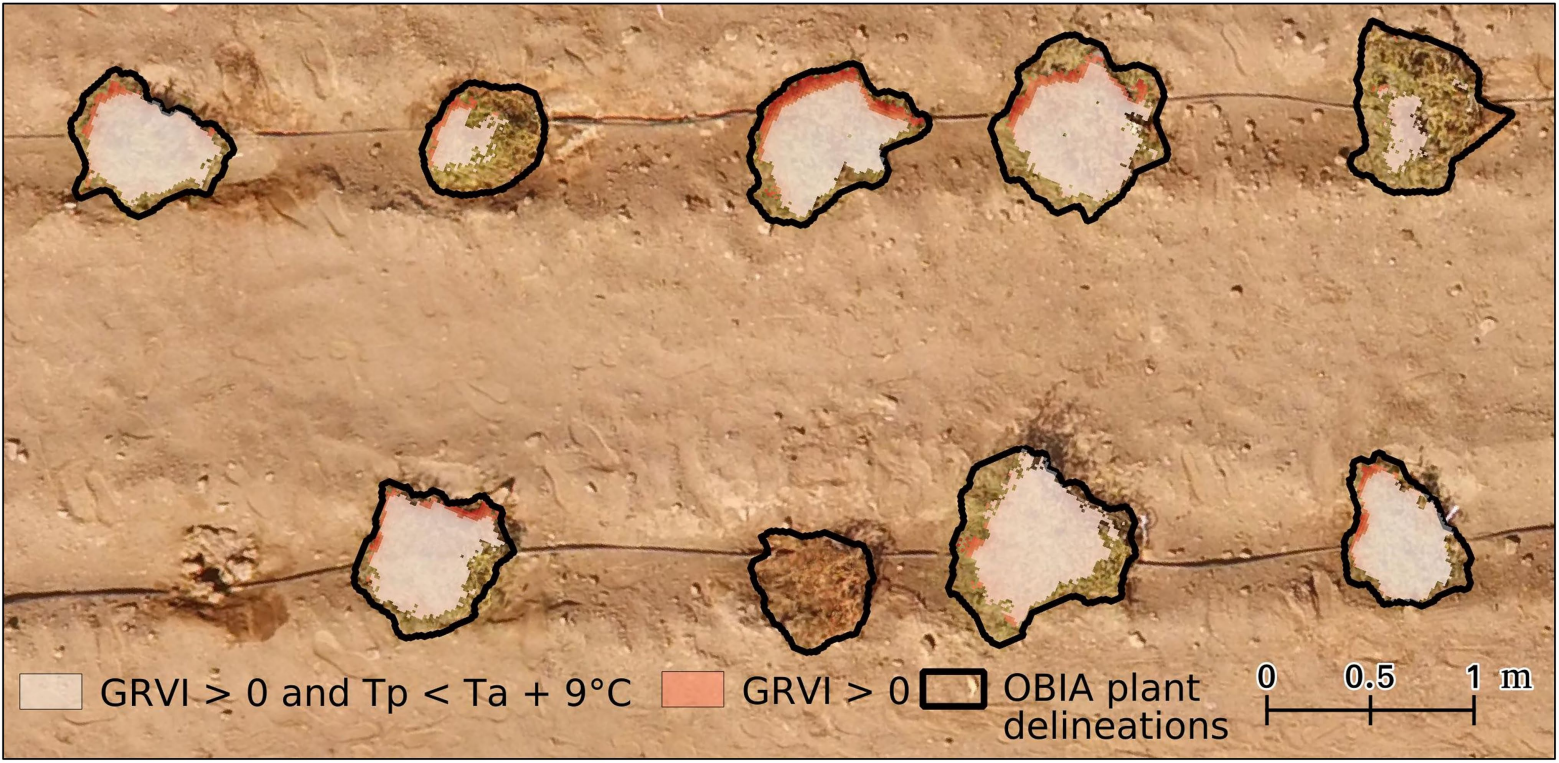
An example of the vegetation mask where the GRVI was greater than 0 (i.e., indicating vegetation) and with pixels greater than air temperature (T_a_)+9°C dropped. Data are overlaid on a red, green, and blue image of six plants in a range of conditions in control plot 2 on January 14, 2018. Note light red on the edge plant corresponds to GRVI > 0 pixels warmer than T_a_+9°C.

Even after the GRVI mask was applied, there were a number of pixels with T_p_ that was considerably higher than that expected for vegetation, indicating mixed pixel or classification issues. Therefore, the approach of Rud et al. (2014) was adopted to determine a realistic estimate for the maximum deviation of T_p_ from T_a_. In this case, a threshold of T_a_+9°C was used after analyzing both the field-installed Apogee radiometer and UAV data for the growing season. Subsequently, any pixels that had positive GRVI values but were warmer than T_a_+9°C were removed to allow the formation of the final vegetation mask, from which T_p_ was ultimately retrieved (see Figures 2, 3).

Following Poblete et al. (2018), a k-mean clustering using a five-cluster *a priori* and k-means++ initialization was also applied on the blue band in order to differentiate sunlit and shaded areas of the tomato plants. The selection of a five-cluster *a priori* was also verified by applying the elbow method to identify the optimum number of clusters (Thorndike, 1953). The maximum blue reflectance value of the first cluster was used as the threshold above which vegetation was identified as sunlit. From the final vegetation mask (Figure 2), we retrieved descriptive statistics of T_p_ (minimum, maximum, mean, median, standard deviation, and pixel count). If the vegetation mask had a pixel count of <10% of the original number of pixels in the OBIA delineation, we removed the plant from further analysis, assuming the plant was dead or that the canopy had senesced and was thus too sparse for accurate T_p_ retrieval.

### Identifying Plant Stress and Calculating Thermal Indices

To consistently compare plant temperature across the five flights, we calculated the deviation of T_p_ from ambient temperature (dT_p_=T_p_ - T_a_), a measure often used in field phenotyping studies of heat tolerance (Balota et al., 2007). To further normalize for meteorological conditions, we calculated the CWSI using Eq (2) (Idso et al., 1981; Jackson et al., 1981), where dT_p_ is the actual difference between T_p_ and T_a_, dT_pLL_ is the lower limit that represents transpiration at the maximum rate (theoretically a non-stressed plant cooled *via* latent heat exchange), and dT_pUL_ is the upper limit that represents a halt in transpiration (theoretically a stressed plant, where sensible heat exchange determines T_p_).


$$CWSI=\frac{{\mathrm{dT}}_{p}-{\mathrm{dT}}_{p_{\mathrm{LL}}}}{{\mathrm{dT}}_{p}{}_{{}_{\mathrm{UL}}}-{\mathrm{dT}}_{p}{}_{{}_{\mathrm{LL}}}}$$ (2)

Traditionally, there have been two ways to derive these transpiration baselines: empirically (CWSI_E_) and theoretically (CWSI_T_), with many researchers reviewing and debating the various limitations of each (Gardner et al., 1992; Maes and Steppe, 2012; Gerhards et al., 2019). The main limitation of the CWSI_T_ is the complex meteorological data required to solve the energy balance equation. CWSI_E_ has seen broad application, as it only needs three variables (T_a_, T_p_, and RH) to be calculated. However, the CWSI_E_ approach requires dT_p_ and VPD measurements to be collected across an entire growing season to calculate robust baselines (Gardner et al., 1992). More recently, UAV studies have proposed a simplified statistical method (CWSI_S_) using the temperature distribution in the image scene to set the baselines (Gonzalez-Dugo et al., 2013; Rud et al., 2014; Bian et al., 2019). This simplified approach is appealing, as it only requires measurements of T_a_, which facilitates applications in precision agriculture (Cohen et al., 2017). However, both stressed and non-stressed plants need to be present in the imagery using the simplified approach.

As our study occurred in Saudi Arabia, where there is a paucity of studies applying the CWSI, we tested all three approaches. For CWSI_E_, we calculated the baselines using the intercept and slope values for tomato plants in Idso (1982). For CWSI_T_, we calculated dT_pLL_ as presented in O’Shaughnessy et al. (2011). As the calculation of dT_pUL_ in CWSI_T_ is error-prone due to the estimation requirements of aerodynamic resistance and roughness length (Idso et al., 1981), we did not calculate it. Instead, we adopted T_a_+9°C as an estimate for dT_pUL_ (see Retrieving Plant Temperature). For the simplified statistical approach (CWSI_S_), we examined the T_p_ histogram distribution and set dT_p LL_ as the mean of the lowest 5% of plant temperatures in the control plots, while dT_pUL_ was set as T_a_+9°C. (Meron et al., 2013; Rud et al., 2014; Bian et al., 2019).

We applied a standard independent two-sample T-test (*α*=0.01) in the SciPy package of the Python 3.5 software language (Virtanen et al., 2020) to assess whether there was a difference in thermal indices between salt-treated and control plots. To understand the change in thermal indices across the season, we calculated the percentage difference between the treatments and plotted the thermal indices as a box plot for each treatment to determine the optimum time to detect stress.

A field-based visual assessment of plants in poor condition was performed on January 4, which identified 30 dead plants. To assess whether T_p_ could be used to identify the dead plants earlier in the season and prior to senescence, 30 healthy plants were also selected from a visual assessment of the January 7 RGB data, with those plants distributed across the two control and two salt plots. That allowed comparison of the plants from the two groups, i.e., healthy and dead in the beginning of January, to determine whether T_p_ could be used for early detection of plant stress, while all plants were still green in December.

## Results

### Discriminating Plant From Soil Temperature in the Thermal Infrared Orthomosaics

**Figure 4:**
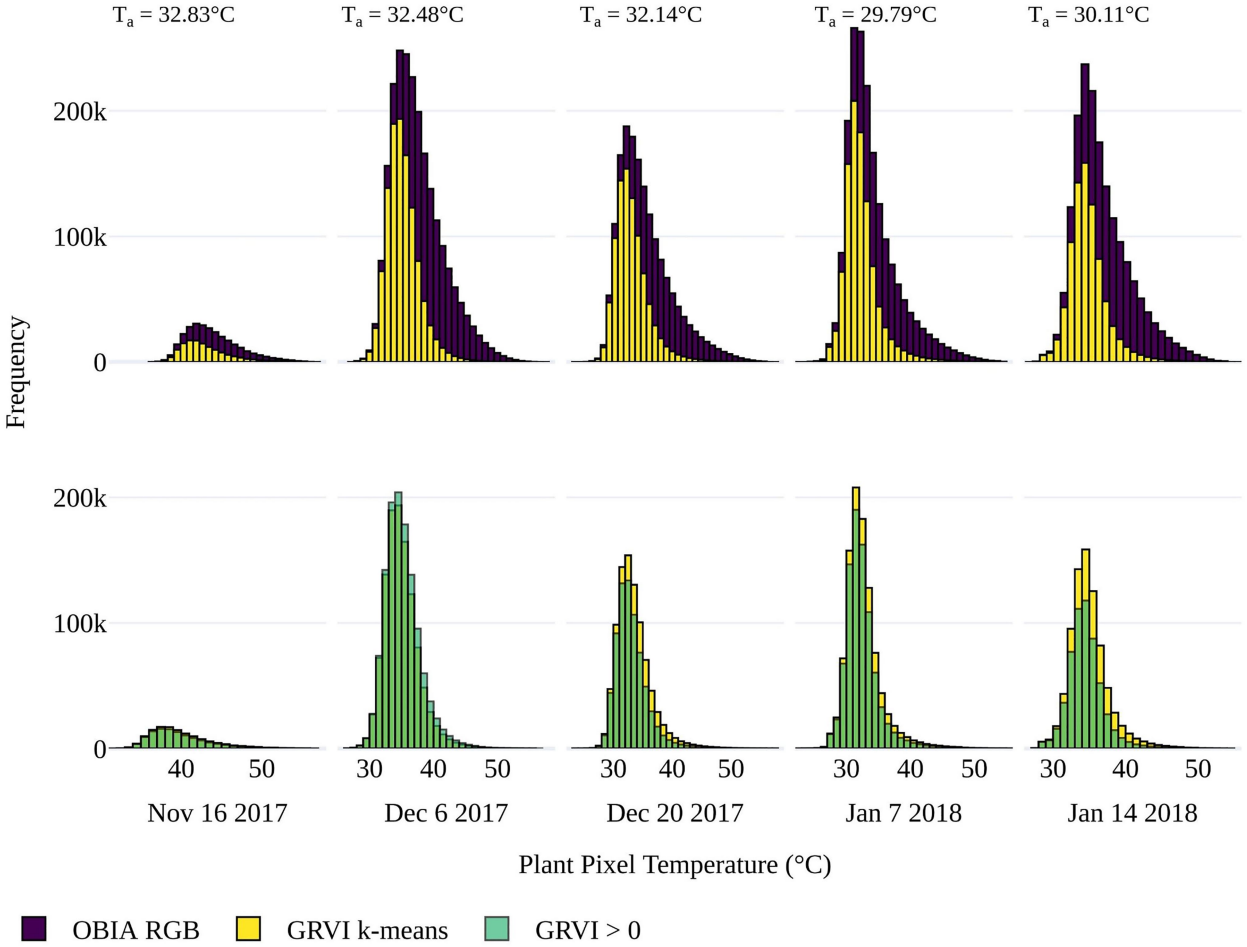
Top Distribution of pixel-based temperatures within the plant delineations from the OBIA approach applied to the red green blue (RGB) data (purple) and for vegetation within the delineations determined by k-means clustering using a two-cluster *a priori* on the (GRVI; yellow) for the five UAV data collection dates. Bottom) The distribution of pixel-based temperature for vegetation classified where GRVI > 0 (green), overlaid on the k-mean approach (yellow) for comparison. When there is a greater frequency of pixels classified as vegetation with GRVI > 0 than the k-means approach (i.e., for December 6), it is shown in a lighter green color. Average air temperature (T_a_) is shown for each flight.

To determine the best approach to discriminate vegetation in the TIR orthomosaics to retrieve T_p_, pixel-based temperature distributions within all tomato plants in the field trial were plotted. The presence of pixel-based temperatures >50°C (i.e., approximately T_a_+20°C) within the OBIA delineations (Figure 4) indicated that some pixels represented soil or non-photosynthetic vegetation. When pixel-based temperature was retrieved using k-means clustering of the GRVI with a two-cluster *a priori* to separate background and vegetation, the frequency of pixels with temperatures >40°C reduced significantly (Figure 4). Therefore, it was assumed that this method was predominantly retrieving temperature from pixels representing vegetation rather than a mixed pixel response. A limitation of the k-means classification was attributed to vegetation being discriminated with a dynamic threshold of the GRVI value for the different campaigns to separate the two classes (Table 2), making a multi-temporal comparison of T_p_ challenging. Using a fixed threshold of GRVI > 0 to discriminate vegetation produced a similar temperature distribution across the five campaigns to that of the k-mean approach (Figure 4). However, the frequency of pixels with positive GRVI values decreased as the percentage of senesced vegetation increased. For example, the k-mean threshold for GRVI that separates vegetation and background was 0.02 on December 6. However, as non-photosynthetic vegetation increased, the threshold became −0.04 by January 14, which was the date exhibiting the largest difference between the two approaches in the number of retrieved vegetation pixels (Table 2). As a consistent comparison across the five flight dates was of most interest, a fixed threshold of GRVI > 0 was adopted for the final mask to retrieve T_p_. However, a flexible clustering approach may produce better discrimination for single campaigns, which can be seen in the reduced number of pixels >40°C in the k-mean approach on December 6 (Figure 4).

**Table 2:** The GRVI k-mean thresholds separating vegetation and the soil background across the five UAV data collection dates, as well as standard deviation (σ) of plant temperature (T_p_) in the field trial for vegetation masks using GRVI > 0 and GRVI > 0 in combination with T_p_<T_a_+9°C.

Flight date	Nov 16, 2017	Dec 06, 2017	Dec 20, 2017	Jan 07, 2018	Jan 14, 2018
GRVI k-mean threshold	−0.02	0.02	−0.03	−0.02	−0.04
Max σ of T_p_ @ GRVI > 0	6.0	7.1	7.8	9.2	4.9
Mean σ of T_p_ @ GRVI > 0	2.3	2.2	2.0	2.1	1.8
Max σ of T_p_ @ GRVI > 0+T_p_<T_a_+9°C	2.6	3.2	3.3	2.7	2.5
Mean σ of T_p_ @ GRVI > 0+T_p_<T_a_+9°C	1.3	1.8	1.6	1.5	1.2

As shown in Figure 4, the number of plant pixels increased through the growing season, peaking on January 7 with a subsequent reduction due to increasing plant senescence prior to harvest. Counter to this trend was the reduction in the number of vegetation pixels on December 20. The fact that this occurred in both the OBIA and GRVI retrievals suggests that the decline may be attributed to the plant damage and decrease in plant area caused by a sandstorm before the UAV capture (Johansen et al., 2019).

**Figure 5:**
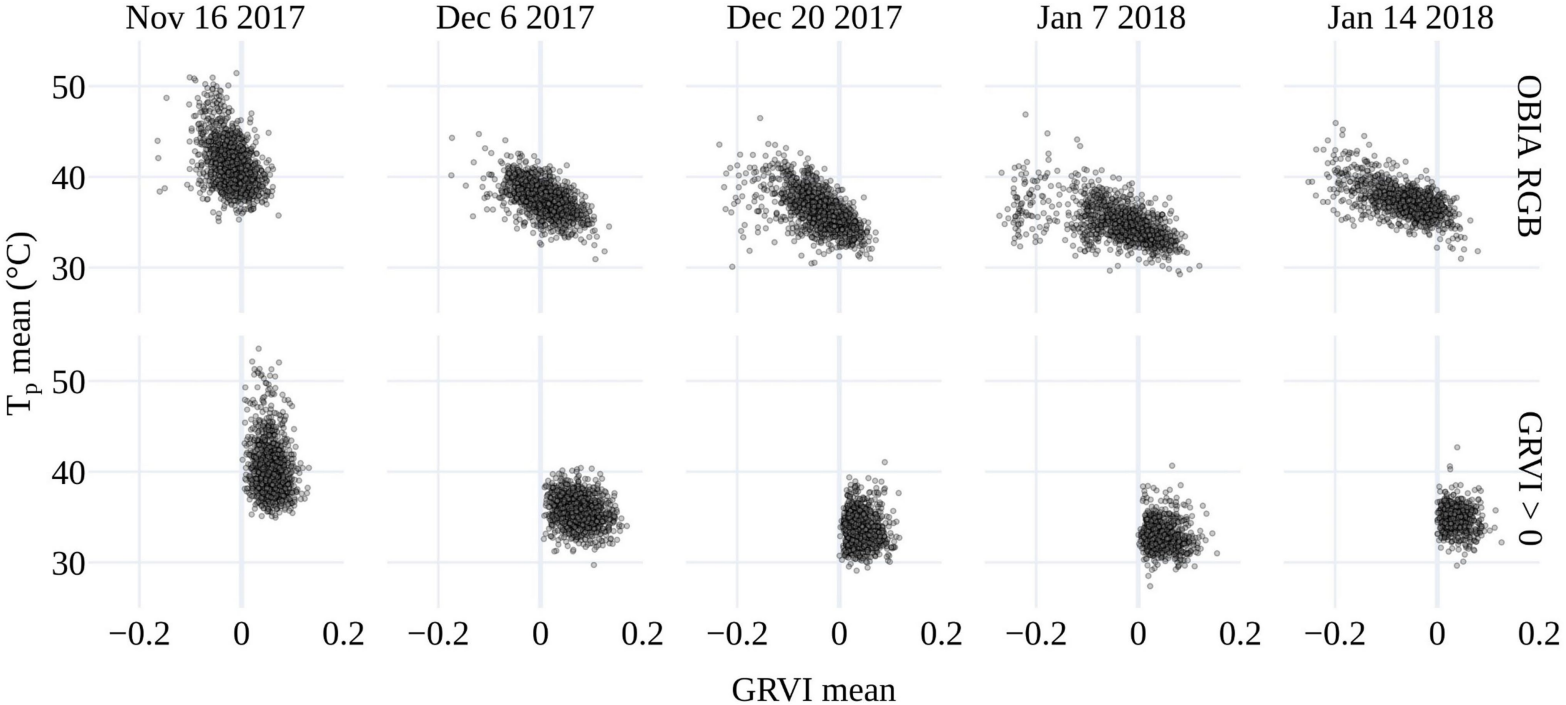
The relationship between mean plant temperature (T_p_) and the mean GRVI for the OBIA delineations (top) and GRVI > 0 retrieval.

There is a tendency toward a negative relationship between GRVI and T_p_, as increased GRVI values (greenness) result in T_p_ decreases due to latent heat exchange during transpiration. In our study, this trend held within the OBIA delineations, which included background soil and non-photosynthetic vegetation (Figure 5). However, there was no clear relationship between T_p_ and GRVI for GRVI > 0. The large range in T_p_ values for pixels with GRVI > 0 and the fact that there were pixels with positive GRVI values that have unrealistically high temperatures for vegetation demonstrated that the GRVI co-registration method did not fully resolve mixed pixel issues. Therefore, we set a more realistic threshold of T_a_+9°C for the maximum deviation of T_p_ from T_a_ to mask pixels further. The need for the T_a_+9°C threshold is shown with the reduction in the maximum standard deviation (σ) of T_p_ before and after the threshold was applied (Table 2). The mean of the maximum σ of T_p_ was 7°C for the five dates with GRVI > 0 but decreased to 2.9°C with the GRVI > 0 and T_p_<T_a_+9°C (Table 2). The drop in the σ of T_p_ indicates that GRVI > 0 and T_p_<T_a_+9°C effectively classified vegetation pixels and omitted background and mixed pixels, which is essential to ensure confidence that changes in T_p_ are an indication of a response to salt stress.

The number of plants from which T_p_ was able to be retrieved with the final vegetation mask (GRVI > 0 and T_p_<T_a_+9°C) compared to the number of plants as identified with the initial OBIA RGB delineation is shown in Table 3. As the growing season progressed, the sample size of the salt and control plots started to differ due to increased deterioration of plant condition in the salt plots based on the GRVI < 0 and T_p_>T_a_+9°C thresholds. Note also that T_p_ was extracted from more plants on December 6 than November 16, due to the small plant size of the initial vegetative growth stage, as well as and soil background effects (i.e., the T_a_+9°C threshold).

**Table 3:** Number of plants for which plant temperature (T_p_) was retrieved in each of the thermal infrared orthomosaics.

UAV flight date	OBIA delineation			OBIA masked for GRVI > 0 &<T_a_+9°C		
	Control	Salt	Total	Control	Salt	Total
November 16, 2017	587	585	1,172	470	464	934
December 06, 2017	587	586	1,173	575	555	1,130
December 20, 2017	583	582	1,165	531	394	925
January 07, 2018	561	566	1,127	490	361	851
January 14, 2018	524	521	1,045	449	251	700

*The number of plants is calculated based on: (1) the delineations of the OBIA approach and (2) the OBIA approach combined with the application of GRVI>0 and T_p_ <T_a_ +9°C*.

#### Examining the Influence of Sunlit and Shaded Components of Tomato Plants

**Figure 6:**
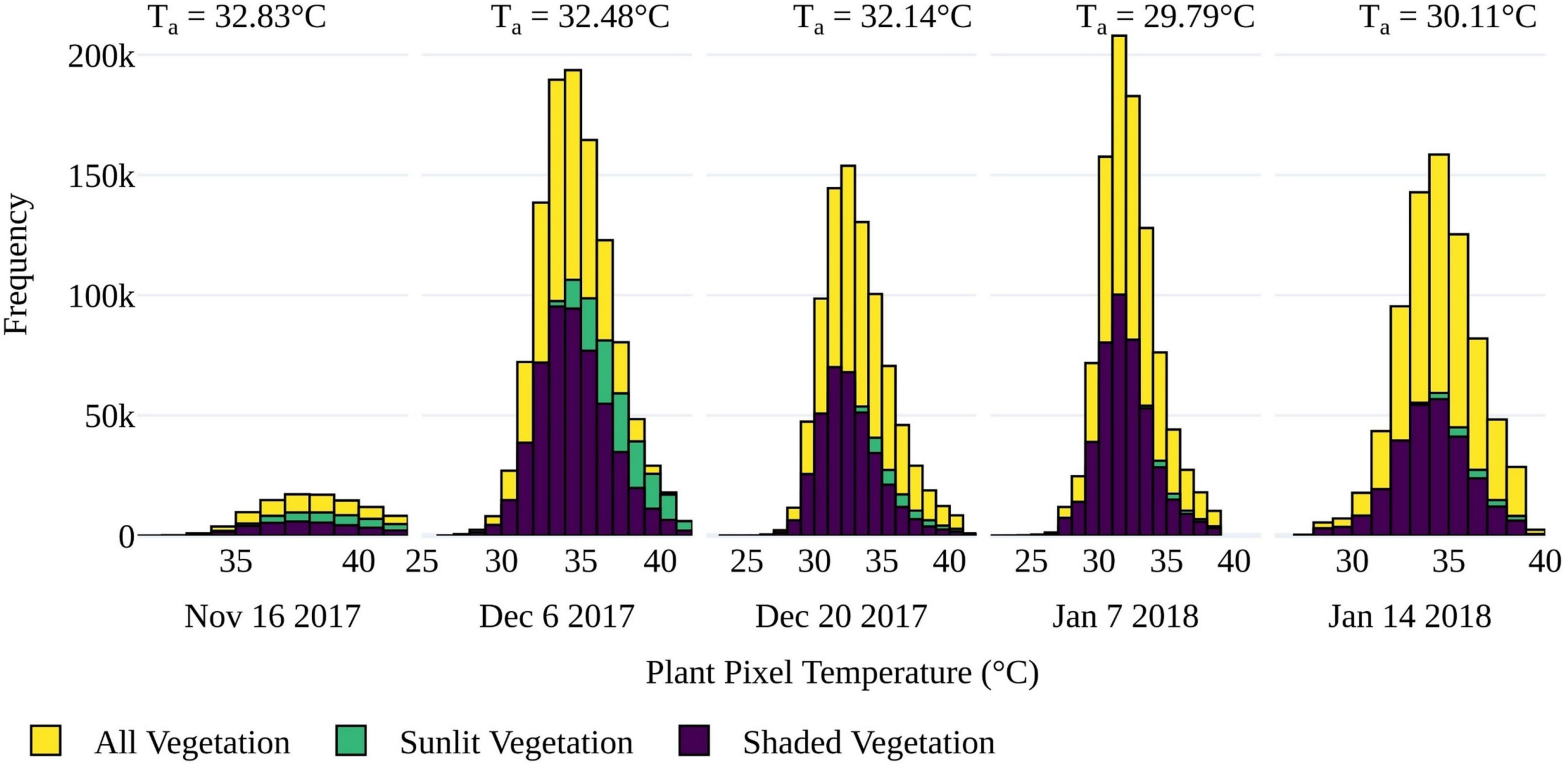
Distribution of pixel temperatures for sunlit, shaded, and all vegetation as classified by GRVI > 0 for the five UAV data collection dates. Sunlit vegetation was identified as pixels with a reflectance value greater than the maximum value in the first cluster of a five-cluster k-mean approach based on the blue band. Average air temperature (T_a_) during each flight is also displayed.

While separating vegetation and soil temperatures is important to minimize mixed pixel responses (McCabe et al., 2008), high-resolution TIR sensing also allows for the discrimination of sunlit and shaded elements within the instrument’s field of view. To assess whether large temperature differences existed between sunlit and shaded vegetation components, the distributions of the sunlit (high reflectance) and shaded (low reflectance) components within the tomato plants (as determined by GRVI > 0) were compared to that of the whole plant, i.e., sunlit and shaded components combined. As shown in Figure 6, the plants had a relatively homogenous temperature range between sunlit and shaded plant components. The largest difference in shaded and sunlit temperatures occurred on December 6, 2017, which coincided with the date of the greenest vegetation (highest GRVI values) and earliest data collection time of 11:00h. The denser, more developed canopy and lower sun angle likely increased the impact of shading on this date. However, as there was no distinct temperature range between sunlit and shaded components, subsequent analysis of retrieved T_p_ of salt stress was based on both sunlit and shaded vegetation, defined by GRVI > 0 and T_p_<T_a_+9°C.

### Can UAV Thermal Infrared Data Identify Stressed Tomato Plants?

**Figure 7:**
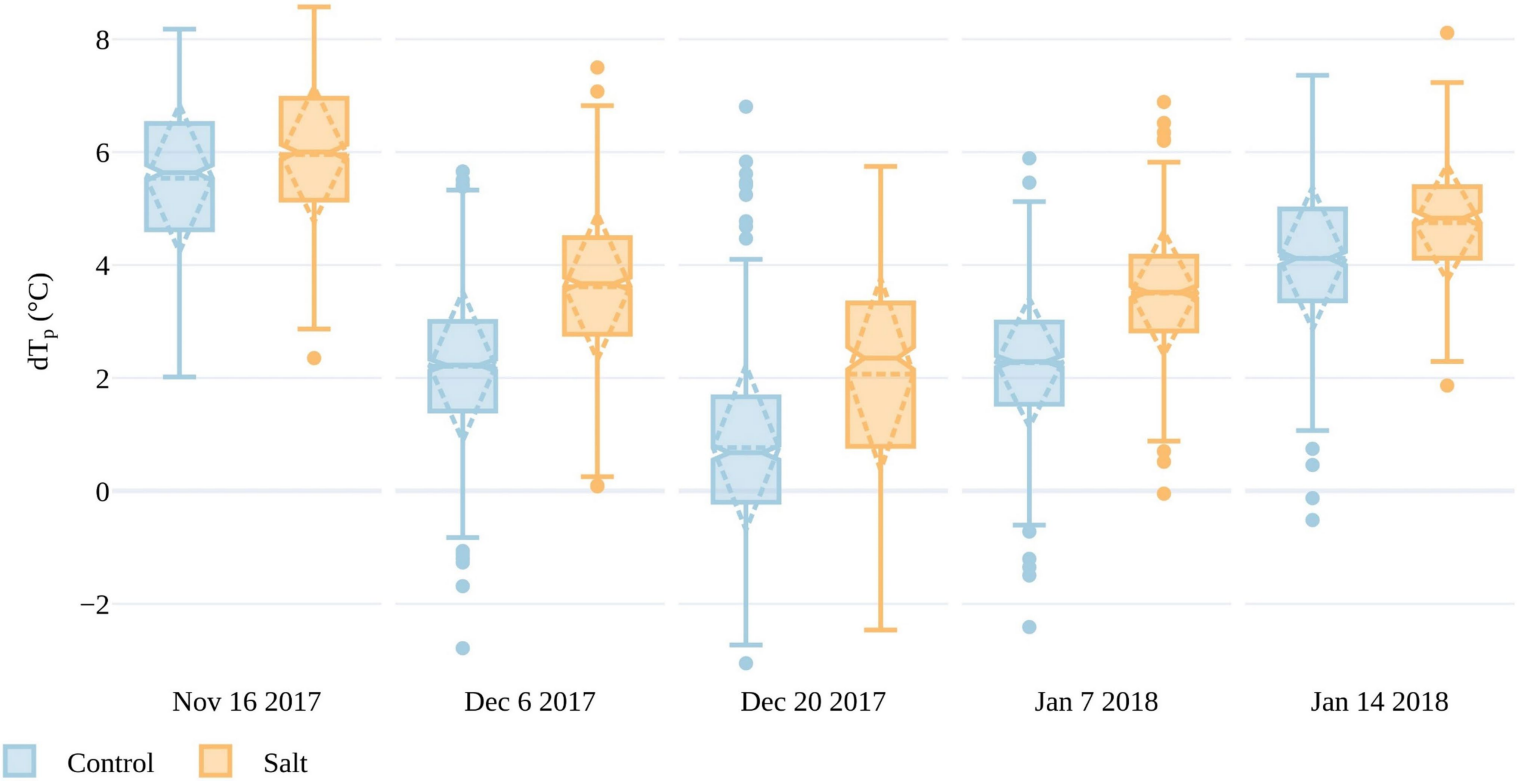
Differences between plant and air temperatures (dT_p_) for all plants within the salt and control plots for the five UAV campaigns. The boxes span the interquartile range (IQR), with notches indicating the median and the dashed diamond the standard deviation and mean. The whiskers bound 1.5*IQR.

To determine differences in plant response to either fresh or saline water irrigation, we assessed the deviation of T_p_ from the ambient temperature in both the salt and control plots. As shown in Figure 7, the mean temperature of tomato plants in the salt-treated plots consistently deviated from the ambient temperature more than the control plots across all five collection dates. The mean dT_p_ was above 5°C in both the salt-treated and control plots during the first collection on November 16, indicating that the plants may have been too small or sparse for accurate T_p_ retrieval. For instance, the mean plant area based on the OBIA RGB delineation was 0.06m^2^ on November 16, but increased to 0.42m^2^ by December 6. From December 6 to January 14, mean dT_p_ increased from 2.2 to 4.1°C in the control plots and from 3.6 to 4.7°C in the salt plots, demonstrating that the salt treatment led plants to have a higher T_p_ above the ambient temperature (Figure 7). The biggest difference in dT_p_ between salt and control plots occurred on December 20, with a difference of 1.3°C. Interestingly, on this day, plants also had the smallest deviation from T_a_, with only one outlier in the control plot exceeding 6°C. The UAV flight on December 20 had a higher VPD (atmospheric demand for water) than on December 6 and January 7 and 14. Often, increasing VPD can lead to an initial increase in stomatal conductance, which decreases as the plant regulates its water exchange (Damour et al., 2010). The influence of VPD on tomato stomatal conductance may have caused the smaller dT_p_ values for this date and may also be contributing to the larger dT_p_ difference between salt-treated and control plants (Patanè, 2011). The difference in dT_p_ between treatments was less apparent on January 14 (4days before harvest), which may have been the result of plant aging and senescence being a larger factor in determining T_p_ than salt stress, as will be discussed in UAV-Derived Plant Temperature.

In order to compare results across the data collections, T_p_ had to be normalized for the variable weather conditions. To do this, the CWSI was calculated in three ways, as presented in Identifying Plant Stress and Calculating Thermal Indices (also see Supplementary Figure S1). Here, we only discuss CWSI_S,_ as it only required measurements of T_a_ and showed similar characteristics to CWSI_E_ and CWSI_T_ (Also, a full season of accurate daily dT_p_ was not available to calculate robust local transpiration baselines.) A smaller difference in CWSI_S_ between the control and salt-treated plots occurred on January 14 compared to the preceding dates. The smaller difference in CWSI_S_ between treatments closer to harvest suggests that T_p_ was better at discriminating stress between the fruit formation and ripening/mature stages (Figure 1), when plants in both plots had more developed canopies. From December 6 to January 7, mean CWSI_S_ in the control plots ranged between 0.23 and 0.27, whereas the salt plots ranged from 0.36 to 0.44, indicating that CWSI_S_>~0.36 may be an indicator of stress. It is worth noting that CWSIs values <0 represent plants that are cooler than the mean of the lowest 5% of plant temperatures in the control plots, which was used to set the lower limit in the CWSI that represents transpiration at the maximum rate. As CWSIs was overestimated if T_p_ was retrieved from non-vegetation surfaces (Irmak et al., 2000), we omitted CWSI_S_ values for November 16 due to large dT_p_ values on that date, which represented T_p_ retrievals integrated the soil background.

**Figure 8:**
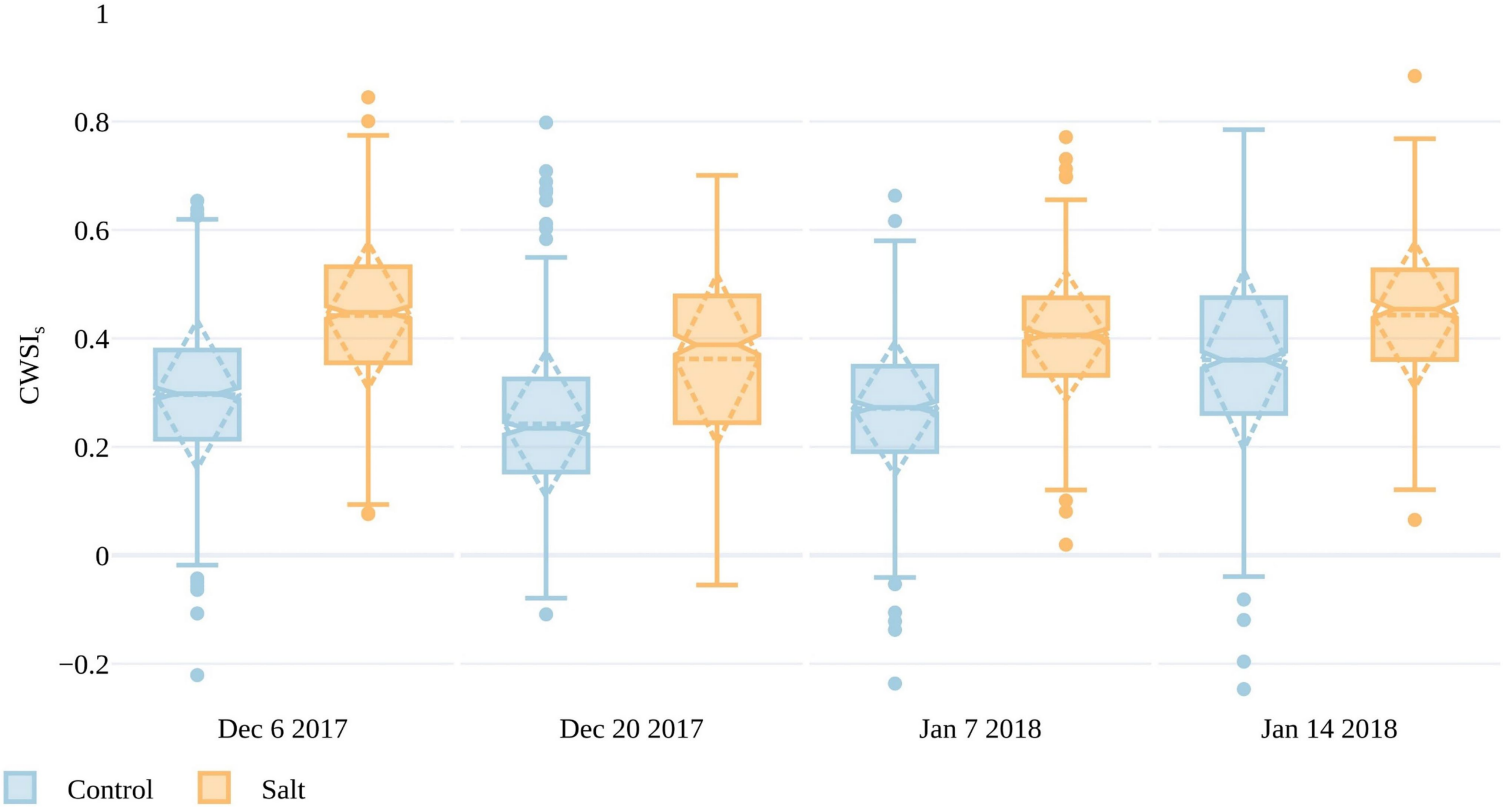
Crop water stress index (CWSI_S_) values for the simplified statistical method over both salt and control plots for four UAV campaigns throughout the growing season. The boxes span the interquartile range (IQR), with notches indicating the median and the dashed diamond the standard deviation and mean. The whiskers bound 1.5*IQR.

**Figure 9:**
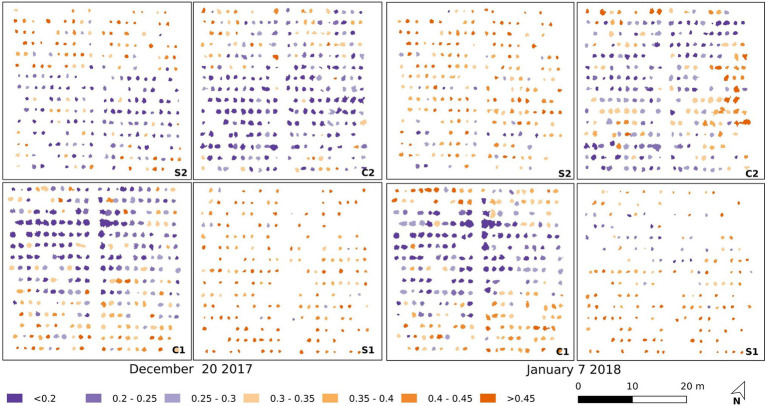
Maps of the CWSIs values for the simplified statistical method in the salt-treated (S1 and S2) and control (C1 and C2) plots for December 20, 2017 **(left)**, and January 7, 2018 **(right)**.

It is apparent from Figures 7, 8 that there is a large range in T_p_ (and consequently dT_p_) and CWSI_S_ values within both the control and salt plots, which may be due to different stomatal responses to stress in each of the 200 accessions, as well as spatial variations within the trial. The spatial variations are plotted in Figure 9, with individual CWSI_S_ shown for both the control and salt treatment for December 20, 2017, and January 7, 2018, which represented the time from fruit formation to mature, ripe fruit. As can be seen, there is a clear tendency for higher CWSI_S_ values in the two salt treatments, relative to the control, with a larger number of plants with CWSIs values >0.35 in the salt-treated plots. For instance, on December 20, only 19% of control plants had a CWSI_S_>0.35, compared to 57% for the salt-treated plants. On January 7, the proportion of plants with CWSI_S_>0.35 for the control and salt plots increased to 24 and 68%, respectively (Figure 9). It is, of course, important to recognize that spatial variability in real-world trials is more than just a function of plant stress, with other soil and environmental factors playing a role. However, while not all aspects of the spatial variation (e.g., the December sandstorms with northeasterly winds) in CWSIs observed in Figure 8 can be attributed to salt-induced stress alone, Figure 9 provides some additional insights to help interpret the influence of irrigation treatments.

**Figure 10:**
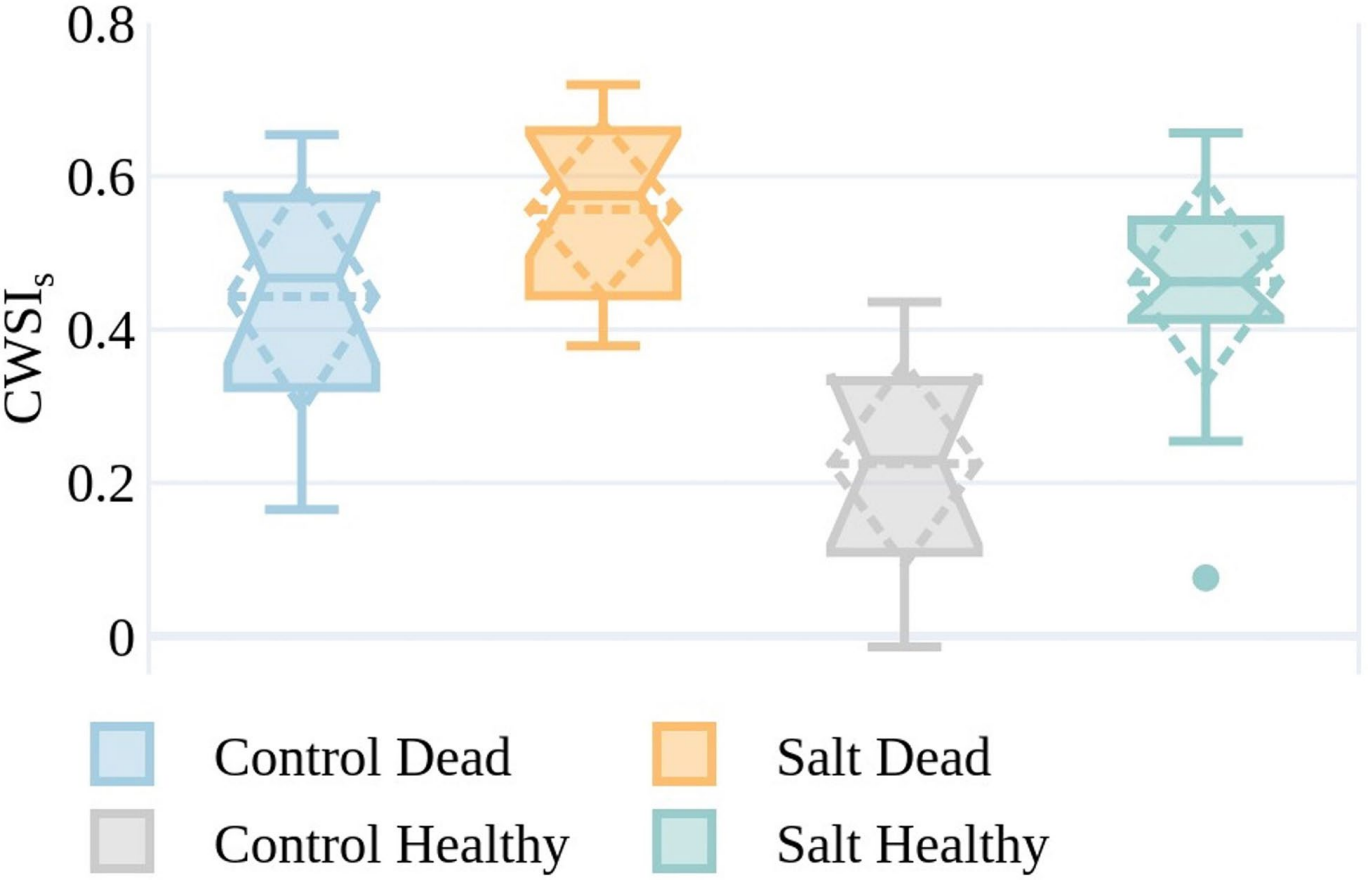
CWSIs values on December 6 for the simplified statistical method for plants from both the control and salt-treated plots identified as either dead (by ground-based visual observation) on January 4 or healthy (by RGB image assessment) on January 7. The boxes span the interquartile range (IQR), with notches indicating the median and the dashed diamond the standard deviation and mean. The whiskers bound the 1.5*IQR. The sample size is reflective of the plants that were identifiable in the UAV imagery with the GRVI > 0 and plant temperature <air temperature +9°C on both December 6 and January 7, or field-identified as dead on January 4 (control dead=12, control healthy=10, salt dead=18, and salt healthy=20).

A field-based assessment of plant condition was undertaken on January 4, with 30 plants identified as dead. An equivalent number of healthy plants were separately identified from the RGB imagery collected on January 7. The CWSI_S_ values for plants in the healthy and dead categories are shown on December 6 in Figure 10 to understand whether CWSIs values measured earlier in the season were indicative of the plant condition in early January.

Plants in the salt plots that were dead by January 4, but in good condition on December 6, generally had higher CWSIs values than those control plants that were still healthy at the beginning of January (Figure 10). Of the plants that were classified as healthy, the ones in the control plots exhibited lower CWSI_S_ values than in the salt plots (median=0.46 and 0.23, respectively). Interestingly to note is that for salt-irrigated plants on December 6, the difference in median CWSI_S_ values between plants that were dead and healthy by the beginning of January (0.57 and 0.46, respectively) is much smaller than for the control plants (0.47 and 0.23, respectively). This is most likely because the salt irrigation caused some level of plant stress early in the growing season, i.e., December 6, irrespective of plant appearance. These differences in CWSI_S_ values on December 6 indicate that at least some plants that appeared green and visibly healthy with GRVI > 0 and T_p_<T_a_+9°C showed early stress warning signs with high CWSIs values almost a month prior to plant death.

## Discussion

Identifying salt-resistant germplasm in field trials is challenging for a number of reasons, not the least being that plant response to stress is complex and manual field methods to screen germplasm are onerous and often subjective (Araus and Cairns, 2014; Morton et al., 2018). UAV remote sensing has emerged to phenotype plants and provides a way to derive an additional understanding of stress responses. Previous research has explored the morphometric detection of salt stress in tomatoes through RBG and multispectral UAV data (Johansen et al., 2019, 2020). While data collection and processing workflows for such approaches are comparatively well developed, the retrieval of accurate T_p_ from UAV TIR data remains challenging (Ribeiro-Gomes et al., 2017; Torres-Rua, 2017; Kelly et al., 2019; Aragon et al., 2020; Perich et al., 2020).

### Challenges in the Retrieval of Plant Temperature From Thermal Infrared Imagery

Here, we explored the retrieval of T_p_ from a UAV TIR camera in a tomato field trial, demonstrating that it is possible to detect differences between salt-treated and control plants, which may help identify salt-tolerant tomato germplasm in future research. In our study, T_p_ was retrieved where GRVI > 0 and setting a maximum pixel threshold of T_a_+9°C. The latter condition was required because the presence of pixels with T_p_>T_a_+20°C in the OBIA plant delineation showed that object-based methods alone are insufficient to retrieve accurate T_p_, at least from the tomato plants explored herein. This finding aligns with Cohen et al. (2017), who also suggest that while object-based approaches work well for tree crops, they fail to retrieve T_p_ from field crops due to their less defined canopy structure. We observed that even when T_p_ is extracted from pixels with GRVI > 0, temperatures that are unrealistically high for vegetation still occurred, demonstrating that the use of GRVI alone does not fully resolve mixed pixel issues. Our findings align with recent UAV TIR studies that could not eliminate all mixed pixels. For example, Zhang et al. (2019) used red and green reflectance together with TIR data to retrieve T_p_ for a maize crop and concluded that better methods for eliminating mixed pixels are required to facilitate accurate extraction.

In our study, the mixed pixel issues were alleviated by combining RGB data with this empirical method (i.e., T_a_+9°C), which estimates the maximum temperature possible for non-transpiring vegetation. Researchers commonly report this empirical upper baseline in studies of drought stress for inclusion in CWSI calculations, e.g., T_a_+5°C in cotton (Cohen et al., 2005), T_a_+7°C in potato (Rud et al., 2014), and T_a_+5°C in wheat (Jackson, 1982) have all been used. The fact that our upper baseline was larger than those published could be attributed to the higher solar radiation and T_a_ of the arid field site or potentially an extreme isohydric behavior (Han et al., 2020), with closed stomata required to maintain turgor. As the field installed Apogee TIR radiometers used for setting the T_a_+9°C threshold make an integrated measurement of T_p_ from their field of view, vegetation movement driven by wind may have occasionally led to the integration of soil temperature, but it was not possible to fully resolve or remove the impact of soil background (Aubrecht et al., 2016).

The successful retrieval of T_p_ using a co-registration approach between the RGB and TIR imagery was dependent on good pixel alignment of the whole study area (Meron et al., 2013). While the datasets in the study were collected with two different sensors (Zenmuse X3 and TeAx 640) having differing resolutions and viewing geometries, they showed good alignment at the GCPs. Future research could identify whether the processing of RGB and TIR data together, as in Javadnejad et al. (2020), leads to better T_p_ retrieval than processing datasets separately with co-registration to GCPs. While new strategies for processing TIR data and identifying vegetation within the orthomosaic would likely improve results, research advances are inevitably constrained by available UAV TIR camera resolutions (640×480 pixels) and precision (Aragon et al., 2020). Although lower flying heights can increase pixel resolution, the downwash from a multirotor UAV may influence measured T_p_ (Tang et al., 2020). Lower flying height also increases flying time to cover the site, increasing the chance of temperature changes occurring during a flight, which could further influence results. The precision of uncooled microbolometers, together with the potential impact of adjacency effects from background scattering (Aragon et al., 2020), adds further uncertainly to derived T_p_ measurements. While the adjacency effect on high-resolution satellite data has recently been explored (Zheng et al., 2019; Duan et al., 2020), the influence on UAV-based data remains under-explored and should be the focus of future work, especially in regard to phenotyping studies, where sub-degree accuracies may be required.

The detection of plant stress *via* UAV TIR data can be sensitive to the level of solar radiation due to its influence on stomatal conductance, with many studies showing the need to consider variation between sunlit and shaded plant components (Jones et al., 2002; Meron et al., 2013; Poblete et al., 2018; Zhang et al., 2019). However, these studies predominately occur in tree or vineyard crops with developed canopies where intra- and inter-plant shading can be significant compared to low profile well-spaced tomato plants. Nonetheless, we examined the temperature difference between high (sunlit) and low (shaded) blue reflectance areas of the plants and found, as opposed to Poblete et al. (2018), that shadowing did not increase the range in T_p_. Therefore, the separation of sunlit and shaded plant components did not improve results in our study. It also meant that methods incorporating the standard deviation of T_p_ as a proxy for transpiration differences between sunlit and shaded areas to detect stress such as in Han et al. (2016), could not be applied to our study.

### UAV-Derived Plant Temperature Can Be Used to Identify Plant Stress

While there are many unresolved questions and inherent sensor limitations for T_p_ retrievals from UAV TIR data, our research demonstrates a detectable difference in T_p_ between the salt-treated and control plots. Differences are apparent across all data collections following the initial salt application on November 14, 2017. Results suggest that T_p_ best discerns plant stress between the stages of fruit formation and ripening (i.e., between December 20 and January 7), an outcome most likely related to canopy cover, which was shown to peak approximately a month before harvest (Johansen et al., 2019). Increased canopy closure reduces soil background influence and increases the plant area over which transpiration is occurring. Once senescence begins, and photosynthesis reduces, and so too does transpiration and canopy cover. This result aligns with Perich et al. (2020), which, although based on a wheat crop, also showed that the optimal time to make TIR measurements is before the onset of senescence. The smaller difference in TIR indices (dT_p_ and CWSIs) between salt and control plots on January 14, together with the broad range in plant condition in both treatments, demonstrates that the morphometric methods of Johansen et al. (2019) present a better approach for identifying stress-tolerant germplasm close to harvest.

Our results suggest that a threshold of CWSIs >0.36 may indicate stress, based on mean differences between salt-treated and control plants and the fact that this threshold applied to 57 and 68% of plants in the salt plot, but only 19 and 24% in the control plots on December 20 and 7 January, respectively. While studies applying CWSI to tomato plants are limited, our results are similar to Anconelli et al. (1993), where CWSI >0.35 led to yield reduction in processing tomatoes (i.e., tomatoes that are canned and machine harvested). Many studies have suggested that CWSI values around 0.3 represent an optimum threshold for commencing irrigation in response to water stress (Reginato, 1983; da Silva and Rao, 2005; González-Dugo et al., 2006). While there are observable differences between the salt and control plots, there is a broad range of dT_p_ and consequently CWSIs values in both treatments. This range may be inherent to the data collection method due to thermal drift or the creation of the orthomosaic. However, compared to previous research we applied a novel orthomosaic generation method by Malbéteau et al. (2021), wherein the temperature of overlapping pixels was averaged along each swath and normalized between-swath temperatures to reduce the impact of standard orthomosaic generation approaches (which integrate overlapping flight lines collected minutes apart and exposed to different wind directions).

Presuming the ranges in CWSIs are reflective of real temperature differences between plants, we suggest that these differences are due to the 200 accessions exhibiting a range of stomatal conductance responses to salt stress. While T_p_ has been used to detect plant stress since the 1960s (Fuchs and Tanner, 1966), it is based on the assumption that plants show an isohydric reaction to stress, reducing stomatal conductance to limit transpiration. A growing body of evidence suggests that plants within the same species exhibit both isohydric and anisohydric responses to stress (Sade et al., 2012). The mechanism employed by tomato varieties with different salt tolerance levels to regulate water use is also unclear (Han et al., 2020). For example, the commercial variety “Moneymaker” (Lycopersicon esculentum Mill., cv) is anisohydric and maintains stomatal conductance in response to stress (Sade et al., 2012). The domesticated variety “Brigade” (Lycopersicon esculentum Mill.) reduces stomatal conductance under drought stress. However, it also opens stomata within a day of irrigation (Patanè, 2011). In comparison, wild types of tomato plants can keep stomata closed for up to 6days after irrigation to maintain turgor (Torrecillas et al., 1995). The variation in stomatal conductance response among the 200 wild genotypes in our trial is still to be determined. Therefore, even with very accurate T_p_ retrievals, cooler plants may not necessarily be the least stressed in terms of agronomically desirable traits such as yield. Plants that had a higher temperature soon after salt application may maintain turgor and produce comparatively higher yields. Resolving this complexity and determining whether T_p_ can be used to differentiate the performance of accessions in our trial are the focus of ongoing research. Identification of inter-accession differences was not the intent of the research presented herein, as the combination of accuracy limitations in current TIR cameras (Kelly et al., 2019; Aragon et al., 2020), the complex role of environmental interactions with plant response, and the uncertainty and complexity in the mechanism employed by *Solanum pimpinellifolium* plants in response to salt stress are all aspects that impact the discrimination of accession-based behavior. Ongoing work will seek to explore some of the genotype–phenotype interactions, and the thermal infrared data may provide some insights into this effort. As UAV-based T_p_ results are confounded by a plant’s morphology (canopy density, leaf inclination), there also needs to be focused research into how to account for morphological variation to increase confidence in the association between observed T_p_ and stomatal conductance (Perich et al., 2020).

The fusion of TIR information with broadband spectral (Johansen et al., 2019) or hyperspectral (Angel, in review) data will likely provide more in-depth insight than TIR data alone to elucidate the challenges in observed T_p_ associated with plant physiological response (Hernández-Clemente et al., 2019). Building upon the results herein and integrating TIR data into the development of turnkey UAV phenotyping solutions could provide a method to enable the early detection of salt impacts by detecting changes in T_p_ in the initial ion-independent response to stress. While our study would have been improved by ground-based visual scoring of plant health during November and December (after the initial salt application), our results showed that CWSI_S_ values were higher in salt-treated than control plants from December 6. Early detection of stress before observed changes in plant form would enable breeders to select germplasm for future breeding studies rapidly and farmers to balance irrigation with brackish water while maintaining yields.

## Conclusion

Salinization is increasingly impacting agricultural land around the world, and available freshwater water resources are increasingly under sustained pressures. Identifying new plant varieties that can either thrive on salinized land or tolerate irrigation with brackish water is crucial to ensuring future water and food security. UAV-based remote sensing has emerged as an effective means to phenotype field plants rapidly. Combining TIR imagery with multispectral data may enable the detection of plant stress before visible symptoms become apparent. Here, we retrieved T_p_ from UAV-based TIR data using concurrently collected RGB data to identify vegetation pixels (GRVI > 0) and an empirical estimate of the maximum possible vegetation temperature (T_p_<T_a_+9°C) to alleviate mixed pixels with background contamination. Results demonstrated measurable differences in T_p_ between salt-treated and control plants across five UAV campaigns performed during the growing season, with analysis suggesting that CWSI_S_ >0.36 was indicative of stress. The reduction in CWSI_S_ differences between treatments toward the end of the growing season demonstrates that the optimum time to use T_p_ for identifying salt stress is between the fruit formation and ripening stages. T_p_ and CWSI_S_ differences between salt and control plots were detectable from December 6, indicating that TIR data may provide a means of early detection of salt stress before visible impacts are discernable. Further research with more frequent image and field data around the initial salt treatment is required to identify the exact time between salt application and a measurable T_p_ response to stress. T_p_ and CWSI_S_ differences were also identified not just between control and salt-treated plants, but between control plants that went on to either die or sustain their plant health a month later. While our analyses provide new insights into the use of UAV-based TIR sensing for the early detection of plant stress, additional research is required to explain both the observed spatial variation and the processes behind stomatal conductance regulation in individual accessions.

## Data Availability Statement

The original contributions presented in the study are included in the article/Supplementary Material, further inquiries can be directed to the corresponding author.

## Author Contributions

BS, YM, and KJ undertook all UAV image processing and analysis. BS led the writing of the manuscript, with KJ, YM, and MM, also contributing. KJ, YM, and MM coordinated field and UAV data collection. KJ carried out the object-based plant delineations. MM designed the UAV-based experiment, including RGB, multispectral, thermal, and hyper-spectral data collection, and was involved in all aspects of the project. All authors contributed to the article and approved the submitted version.

## Funding

MT and his team were supported by the King Abdullah University of Science and Technology (KAUST) Office of Sponsored Research (OSR) under Award No. 2302-01-01 for undertaking the plant experiments. MM and his team were supported by Competitive Research Grant Nos. URF/1/2550-1 and URF/1/3413-01 for undertaking the UAV-based component of this research.

## Conflict of Interest

The authors declare that the research was conducted in the absence of any commercial or financial relationships that could be construed as a potential conflict of interest.

## Publisher’s Note

All claims expressed in this article are solely those of the authors and do not necessarily represent those of their affiliated organizations, or those of the publisher, the editors and the reviewers. Any product that may be evaluated in this article, or claim that may be made by its manufacturer, is not guaranteed or endorsed by the publisher.
