# Supplementary material for: Detecting Plant Stress Using Thermal and Optical Imagery From an Unoccupied Aerial Vehicle
Source: Front Plant Sci. 2021 Oct 27;12:734944. doi: 10.3389/fpls.2021.734944 (PMC8579776; doi:10.3389/fpls.2021.734944)
Supplement: Supplementary file 1 [file Table_1.DOCX]

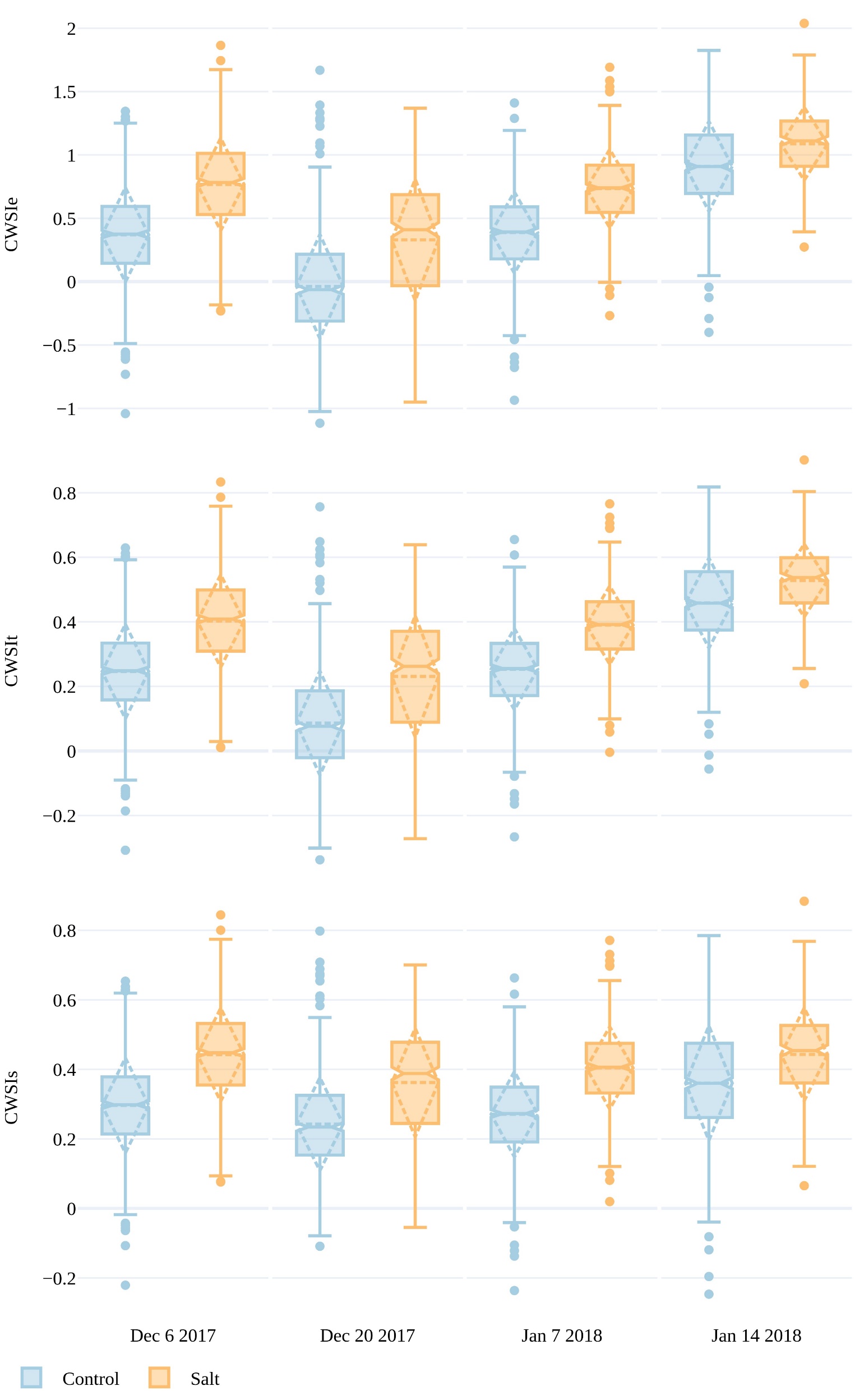


Supplementary Figure 1: Crop water stress index values for three methods: empirically (CWSIe), theoretically (CWSIt) and simplified statistical (CWSIs) over both salt and control plots for four UAV campaigns. The boxes span the interquartile range (IQR), with notches indicating the median and the dashed diamond the standard deviation and mean. The whiskers bound 1.5*IQR.
